# Reverse Transcriptase Inhibitors Nanosystems Designed for Drug Stability and Controlled Delivery

**DOI:** 10.3390/pharmaceutics11050197

**Published:** 2019-04-27

**Authors:** Fedora Grande, Giuseppina Ioele, Maria Antonietta Occhiuzzi, Michele De Luca, Elisabetta Mazzotta, Gaetano Ragno, Antonio Garofalo, Rita Muzzalupo

**Affiliations:** Department of Pharmacy, Health and Nutritional Sciences, University of Calabria, Via P. Bucci, 87036 Rende (CS), Italy; mariaantonietta.occhiuzzi@unical.it (M.A.O.); michele.deluca@unical.it (M.D.L.); mazzotta-elisabetta@libero.it (E.M.); gaetano.ragno@unical.it (G.R.); antonio.garofalo@unical.it (A.G.); rita.muzzalupo@unical.it (R.M.)

**Keywords:** HIV, antiretrovirals, nanoformulations, drug degradation, drug protection

## Abstract

An in-depth analysis of nanotechnology applications for the improvement of solubility, distribution, bioavailability and stability of reverse transcriptase inhibitors is reported. Current clinically used nucleoside and non-nucleoside agents, included in combination therapies, were examined in the present survey, as drugs belonging to these classes are the major component of highly active antiretroviral treatments. The inclusion of such agents into supramolecular vesicular systems, such as liposomes, niosomes and lipid solid NPs, overcomes several drawbacks related to the action of these drugs, including drug instability and unfavorable pharmacokinetics. Overall results reported in the literature show that the performances of these drugs could be significantly improved by inclusion into nanosystems.

## 1. Introduction

The human immunodeficiency virus (HIV), belonging to the lentivirus genus of the large family of retroviridae, is the etiological agent of AIDS. The infection causes severe consequences to the immune system including a loss of CD4+T lymphocytes that leads to an increased susceptibility to even fatal opportunistic infections. The identification of various antiretroviral drugs allowed defining efficacious therapeutic regimens for the prevention and treatment of the disease by the combined administration of two, three or more different drugs acting on crucial steps of viral replication. In particular, the targets of conventional drugs are proteins involved in the viral entry or specific enzymes necessary for the virus replication such as protease (PR), reverse transcriptase (RT) and integrase (IN). This approach known as HAART (highly active antiretroviral therapy) nowadays represents the most useful therapeutic treatment, even though it is affected by many drawbacks such as lifetime administration with a consequent reduced patients’ compliance, severe side effects, and quick viral outbreak after drug resistance emergence. HAART was demonstrated to be particularly effective in cutting down the overall number of viral particles, but is unable to completely eradicate infection in sanctuary sites, such as the brain, liver and lymphatic system [1,2,3,4,5]. Moreover, HAART always includes one or more nucleoside and non-nucleoside reverse transcriptase inhibitors (NRTI and NNRTI, respectively), which, despite a high antiviral efficacy, unavoidably show important clinical drawbacks. Relevant information on common RTI is summarized in Table 1 [6,7]. 

The above therapeutic strategy is not even capable of stimulating a lasting immune response of memory cells necessary to antagonize the infective agent [8]. Even after taking into account all these considerations, innovative therapeutic approaches based on both the identification of alternative drugs and innovative pharmaceutic formulations still have demanding requirements [9,10,11]. Nanotechnologies could help to reach this latter crucial point in order to increase cellular uptake, enhance drug distribution, prolong half-life and reduce side effects depending on the lower drug dosage in the nanosystem. In particular, application of nanoformulations consisting of a given drug and a supramolecular matrix such as niosomes, liposomes and solid lipid nanoparticles (SLN), already led to some improvements of pharmacokinetic and pharmacodynamic parameters. Very interesting results have been recorded in the anti-cancer research field where an altered microenvironment of cancer cells facilitates a selective drug delivery [12]. A similar approach could be adopted in the case of cells infected by HIV [13]. 

**Figure 1:**
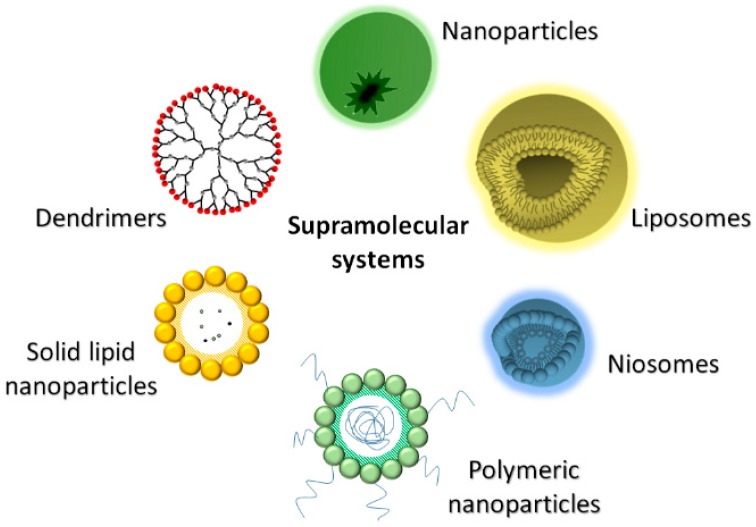
Drug delivery systems for RTI nanoformulations.

In the light of these findings, the incorporation of new or customary anti-HIV drugs into supramolecular carriers could be particularly effective in suppressing viral replication. This strategy is corroborated by the possibility of encapsulating drugs or genes to not only be delivered next to the infected cells but also to target reservoir tissues to eradicate latent HIV [14]. This innovative strategy is suitable for improving the distribution of both hydrophilic and hydrophobic small molecules, as well as macromolecular drugs, which can be driven toward specific tissues thanks to the reduced size of the nanoscale delivery systems. Antiretroviral drugs can be carried as nanoparticles for their potential to better reach macrophages, CD4+T cells and latent reservoirs organs, such as brain and lymph nodes, that are particularly responsible of viral survival. [15,16,17,18,19]. Drug delivery systems (DDS) for RTI, developed in the last few years, are described in this survey and depicted in Figure 1, while their main properties are summarized in Table 2. 

**Figure 2:**
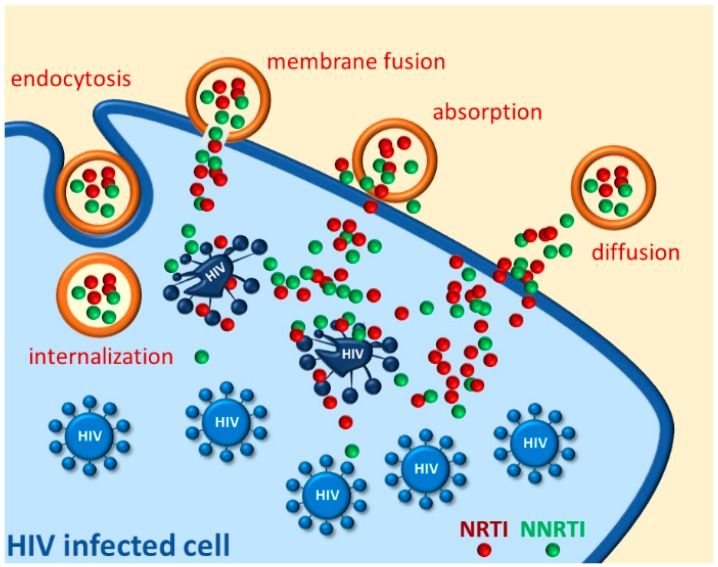
Representative delivery modalities for NRTI/NNRTI nanosystems to HIV reservoirs.

Once the supramolecular system reaches the site of action, a controlled drug release provides high local concentration and longer residence time, resulting in an improved antiviral effect (Figure 2)**.** Furthermore, some nanomaterials possess themselves favorable biological effects. The nanotechnology approach has been advanced for many aspects dealing with HIV infection, namely theranostic, vaccine prophylaxis and gene therapy. This survey however focuses on studies describing NRTI and NNRTI based nanoformulations for prevention or treatment of HIV infection. Although these systems allow a remarkable improvement of the pharmacokinetics and pharmacodynamics of RTI, several drawbacks still need to be overcame. The major advantages and limitations of known nanosystems are listed in Table 3.

Several of these clinically used drugs show an undesirable stability profile when exposed to stressing conditions either in solution or in solid form [20,21]. Similarly, stress tests have been performed on supramolecular systems in order to confirm any improved stability, under different conditions [22]. In particular, studies published in the last few years on the RT inhibitors included in combined therapy have been taken into consideration [15,16,23].

## 2. Drug Protection Nanosystems

Most of the protocols adopted for studying the drug stability are suggested by the ICH (International Conference on Harmonization) guidelines [24]. According to such rules, clearly defined drug storage conditions are required to prevent the degradation effects related to pH, temperature, light, air and humidity for either solution/suspension or commercial formulations/packaging [25]. The protection of sensitive drugs could often be assured by shielding with an adequate packaging [26]. When this simple precaution showed not to be sufficient, the stability of the drug, exposed to different environmental conditions, could be improved by suitable delivery devices, such as vesicular matrices (i.e., liposomes and niosomes), nanoparticles (NPs), and solid lipid nanoparticles (SLN).

Niosomal and liposomal vesicles consist of amphiphilic molecules and an aqueous compartment and differ for their structural chemical units. Both systems are very versatile. The hydrophilic drugs can be entrapped in their aqueous core while the lipophilic drugs can be partitioned into the bilayer domains. Liposomes are made of natural phospholipids, resulting in a greater stability, a low production cost and reduced toxicity [27,28,29,30,31,32], while niosomes are prepared by means of synthetic, non-ionic surfactants, as alkyl ethers, alkyl esters and pluronics copolymers, or fatty acid and amino acid compounds [31,32,33]. The preparation of these vesicles requires a simple procedure based on a gentle agitation or sonication of an aqueous solution of phospholipid/surfactant and drug mixtures taken from an ultracentrifugation or low-pressure gel filtration chromatography to purify the formed systems [34]. 

More recent SLN have been proposed as alternative formulations for both hydrophilic and hydrophobic drugs. These systems are colloidal carriers based on a solid phase lipid and a surfactant and are characterized by a spherical shape in which the lipid portion is always solid and the surfactant acts as a stabilizing factor [22,35,36,37,38,39,40]. Fatty acids, monoglycerides, diglycerides, triglycerides, waxes and steroids can be applied in the preparation of SLN in the absence of organic solvent [35]. Low cost, good physical stability, large scale production, no toxicity and high biodegradability represent the greatest advantages in the use of these formulations with respect to the liposomes matrices [41]. 

Nanoparticles systems are known promising carriers for the improvement of solubility and pharmacokinetics of drugs as well as vaccines, nucleic acids and therapeutic proteins. These delivery devices can influence therapeutic efficiency of a drug, enhance its protection from degradation and reduce dose-limiting side effects. A variety of hydrophobic or hydrophilic active molecules can be dissolved, encapsulated, absorbed or conjugated to polymeric nanoparticles following different techniques [42]. Several natural and biodegradable materials like chitosan have been proposed for the realization of anti-HIV drug nanosystems. An alternative approach, based on the formation of crystalline complex with a fixed range size, was attempted by inclusion of the pure drug into a hydrophobic synthetic polymer [43]. Polymeric nanoparticles based on poly(lactic acid) (PLA) or poly(lactide-*co*-glycolide) (PLGA) are reported as ideal delivery systems, showing an improved therapeutic efficacy with lower incidence of side effects [42,44,45,46,47,48,49]. 

A higher local concentration of active molecules is often reached by integration of classic antiretroviral drugs in different NPs of metals [50]. According to a relative inertness and low toxicity, silver or gold NPs have been explored in biomedicine as multifunctional scaffolds. In particular, the application of gold NPs has been employed to conjugate biomolecules on the outer surface. Alternative inorganic multifunctional materials, such as silver NPs coated with poly(vinyl)pyrrolidone, have also been exploited as drug carriers. [51]. 

Synthetic well-defined nanopolymers with a three-dimensional architecture, known as dendrimers, have been proposed for the vehiculation of several drugs. Generally, a dendrimer is a symmetric and hyper-branched macromolecule characterized by the presence of reactive groups in the central core, repeated branching units in the interior layers of the core and functional groups spanning from the outer surface. The drug molecule can be either entrapped inside the structure or linked to the external functional groups. This approach was proposed for the carrying of several anti HIV agents [52,53,54,55]. 

Some of these matrices are also able to improve the stability of drugs. Several studies described the use of niosomes, metal based and polymeric NPs to prevent the degradation effects caused by stressing conditions [33,50,56].

## 3. Nucleoside Reverse Transcriptase Inhibitors Nanosystems

**Figure 3:**
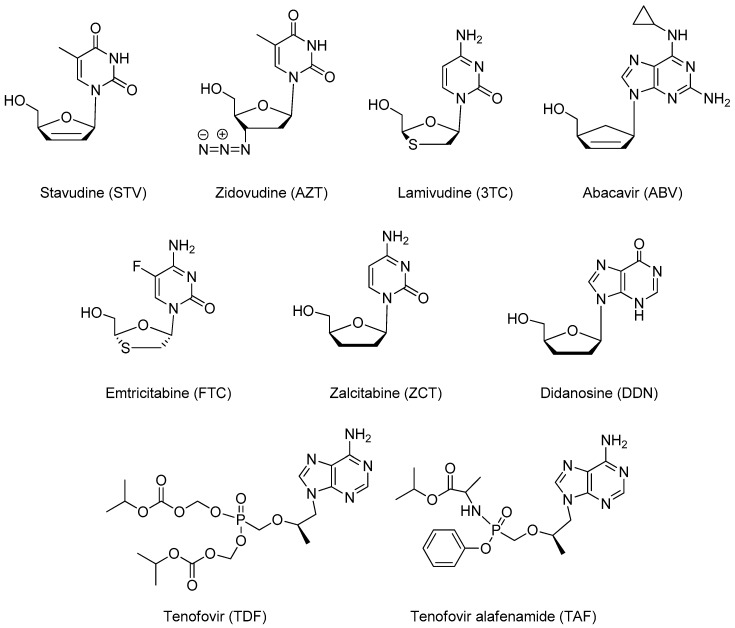
Chemical structures of NRTIs.

The therapy based on the administration of nucleoside antiviral derivatives such as stavudine, zidovudine, lamivudine and emtricitabine represented a first and effective approach adopted for the management of HIV infection (Figure 3). 

**Figure 4:**
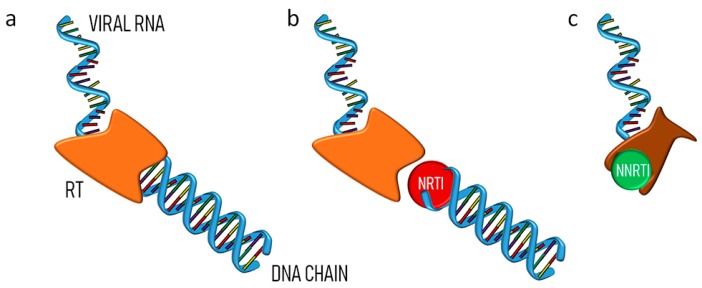
(**a**) RT catalyzes the conversion of viral RNA into pro-viral DNA before its incorporation into the target cell genome; (**b**) NRTIs are incorporated into the DNA causing chain termination; (**c**) NNRTIs bind the enzyme inhibiting its function.

Due to the structural similarity to purine or pyrimidine nucleosides, the mode of action of these drugs consists of the competition for the incorporation into viral DNA, catalyzed by RT, so causing chain termination (Figure 4, panel b). Nowadays the most common nucleoside derivatives used in therapy are lamivudine, abacavir, emtricitabine, tenofovir and tenofovir alafenamide [57]. Stavudine and zidovudine, not yet recommended in first-line therapy, were early examples of NRTIs included in nanoformulations designed for limiting their severe side effects and improving pharmacokinetics.

### 3.1. Stavudine

Stavudine (1-((2R,5S)-5-(hydroxymethyl)-2,5-dihydrofuran-2-yl)-5-methylpyrimidine-2,4(1H, 3H)-dione, STV) is one of the most commonly used forms of NRT approved by FDA in 1996 and its use was recommended in association with other antiretroviral agents. The drug showed major side effects, such as high blood lactate, pancreatitis and hepatomegaly. STV was characterized by a serum half-life of 1h only, while that of its phosphorylated active metabolite was calculated as being 3.5 h [27]. Thus, STV loaded formulations able to concomitantly increase cellular uptake and sustain release should reduce unwanted effects. Accordingly, galactosyl or mannosyl coated liposomes loaded with STV were described. These formulations reached the desired results showing an increased *in vitro* anti-HIV activity together with a remarkable decrease of side effects. The efficacy was tested in a mononuclear phagocyte system, a major reservoir of HIV, proving advantages in terms of bio-stability, site-specific and ligand-mediated delivery, compared to free drug and uncoated liposomes [27,28]. More recently, STV-containing nanoformulations were proposed for the dual utilization to control the residual viremia as well as to target the reservoir sites. To achieve this aim, gelatin nanoformulations containing very low dosage of the drug were prepared through a simple desolvation process and loaded into soya lecithin based liposomes [29]. A study on STV degradation under different stress conditions (hydrolysis, oxidation, photolysis and thermal stress) was initially reported. A stability-indicating reversed-phase HPLC assay method showed the hydrolysis of the drug to thymine in acidic, neutral, alkaline and under oxidative stress conditions [20]. In order to improve the stability of this drug, STV-loaded SLN for intravenous injection were produced by high-pressure homogenization of drug lipid melt dispersed in hot surfactant solution [22]. This SLN formulation was also studied for its active delivery to lymphatic tissues by ex vivo cellular uptake evaluation in macrophages. Reported experiments confirmed an improved cellular uptake together with a prolonged activity next to the delivery site of the formulation compared to the simple drug solution. This could account for an efficient and safe therapeutic profile of the drug-carrier system [58]. 

### 3.2. Zidovudine

Zidovudine, also known as azidothymidine (1-((2R,4S,5S)-4-azido-5-(hydroxymethyl)tetra hydrofuran-2-yl)-5-methylpyrimidine-2,4(1H,3H)-dione, AZT), the first antiretroviral medication proposed to prevent and treat HIV/AIDS, has been approved in 1986. An extensive first pass metabolism often requires an in vein administration. This feature and a long list of severe side effects limit the use of this drug, which is however still present in many therapeutic anti-HIV regimens. Its incorporation into supramolecular matrices was extensively exploited in order to increase bioavailability and to reduce dose-dependent unwanted effects. 

Positively and negatively charged liposomes based on stearylamine and diacetyl phosphate were used as AZT carriers. In order to enhance localization to lymph nodes and spleen, these systems were even coated with a site-specific mannose-terminated stearylamine ligand. Fluorescent microscopy images showed an enhanced uptake and localization of these liposomes in the target tissues [59]. In an early paper, a dispersed system comprising polyoxypropylene, polyoxyethylene, oleic acid, water and cetyl alcohol as surfactant, was described as a potential DDS. The release profile experimental analysis showed that the delivery of AZT could be controlled this way, in accordance with a mathematical theoretical approach [60]. This system has been proposed as a carrier, which potentially could overcome the main drawbacks of conventional pharmaceutical formulations [61]. 

AZT loaded in polymeric NPs based on PLA and poly(l-lactide)poly(ethyleneglycol) (PLA/PEG) were prepared by double emulsion solvent evaporation and thoroughly investigated *in vitro* for uptake into polymorphonuclear leucocytes of rat peritoneal exudate. The cells activation by NPs was assessed by a chemiluminescence assay suggesting a more favorable behavior of PLA vs. PLA/PEG complexes [62]. On the other hand, the drug release increased proportionally to the PEG amount in the blend [63]. AZT was encapsulated in alginate-glutamic acid amide based NPs obtained by an emulsion solvent evaporation method. The polymeric NPs were coated with pluronic F-68 to favor cellular internalization through the endocytosis mechanism. As a result, the antiviral drug loaded in these nanosystems was released in a prolonged manner. Intracellular uptake and cell viability assays also confirmed an efficient uptake of AZT in glioma cell lines [64]. Solid lipid NPs based on modified stearic acid and *Aloe vera* extract were described as an alternative drug delivery carrier for controlled release and targeting of AZT. The plant extract was used because of its high content of polysaccharides that showed synergistic antiretroviral activity with AZT. The described nanocarriers did not interact with plasma proteins and showed high drug loading and entrapment efficiency. Moreover, fluorescent microscopy images suggested that the natural gel facilitated the cellular uptake of AZT in brain cells [36]. The drug proved to decompose when exposed to light or under hydrolytic conditions, while it was more stable toward oxidation agents and thermal stress [65]. In particular, the acid degradation induced the formation of a pyrimidine derivative endowed with higher toxicity when compared to AZT, as demonstrated by a mutagenicity and an aerobic biodegradability assay [21]. Recently, three novel prodrugs of AZT, obtained by functionalization with dicarboxylic acids, were designed in order to enhance pharmacokinetics, chemical stability and affinity for human serum albumin [66].

### 3.3. Lamivudine

The oral agent lamivudine, (4-amino-1-((2S,5R)-2-(hydroxymethyl)-1,3-oxathiolan-5-yl)pyrimidin-2(1H)-one, 3TC), an analog of nucleoside cytidine, was approved by FDA for the combined therapy with AZT in 1995 and for monotherapy in 2002. However, the emergence of drug resistance, associated to the gene mutation of RT, limited its clinical application [67,68]. 

Several nanocarriers were prepared by mixing biodegradable networks (i.e. PEG, pluronic-polyethyleneimmine (PEI), glycyrrhizin conjugated chitosan, mannosylated-PLGA) or dendritic networks (i.e. starPEG-PEI, poly(amidoamine)dendrimer-PEI-PEG) or nanogels with AZT or didanosine triphosphates under a freeze-drying method, to specifically deliver the antiviral agent next to macrophages in the CNS.

All nano-NRTIs demonstrated high efficacy in inhibiting HIV at low µM drug concentration. The major cause of NRTI neurotoxicity, consisting in the mitochondrial DNA depletion, was also reduced 3-fold compared to uncoated NRTIs [69]. Acid or alkaline conditions as well as an oxidative environment caused the degradation of the drug into five different products. On the other hand, light exposure or thermal stress did not affect drug stability [70,71]. 

More recently, a mass spectrometry study evidenced the formation of an additional degradation product when the solid drug was exposed to oxidative conditions [72]. 3TC was incorporated into polymethacrylic acid NPs in different drug/polymer ratio by nanoprecipitation method in order to overcome some drug limitations, such as accumulation during multi dose therapy and poor patient compliance. These polymers offer several advantages, including high stability and simple preparation route compared to remaining colloidal carriers. Moreover, these nanocarriers were shown to increase drug bioavailability and optimize the release time to the target site without significant chemical interactions between the drug and matrix [73]. An alternative encapsulation for 3TC was exploited by using PLGA NPs coated with bovine serum albumin through a double emulsion procedure. It was then demonstrated that PLGA NPs were rapidly internalized into the human liver cells after oral administration, at different drug concentrations, confirming their high potential as ideal 3TC delivery systems [74]. The PLGA/3TC system was also investigated for the formulation of a thermosensitive vaginal gel. The system was obtained in the form of NPs by the formation of an amide bond between the biodegradable polymer and the free amine group of the drug. An analogue formulation was prepared using emtricitabine as an alternative NRTI. The NPs were finally incorporated into a thermosensitive gel for vaginal administration. The nanoformulations showed to be non-toxic in HeLa cells assay up to a 100 µg/mL concentration. Similar preparations containing fluorescent NPs were found to be active for up to 5 days, suggesting a potential long-lasting application in therapy [75]. A similar approach was adopted for the achievement of transdermal formulation of PLGA/3TC complex. NPs obtained resulted in an ideal spherical shape and an external smooth shell. The high drug entrapment rate resulted in an improved physical stability of the drug together with an efficient delivery after skin permeation. This last property was enhanced after microneedles skin pre-treatment [76]. Mannosylated-PLGA NPs were prepared to ensure an efficient delivery of 3TC into brain macrophages. The experimental data confirmed the increased drug release from nanocarriers and this effect may be due to the presence of sugar receptors on the luminal surface of blood-brain barrier (BBB) [77]. 3TC was also entrapped into PLA/chitosan (CS) NPs by an emulsion technique. The drug was efficaciously entrapped and protected at low values of pH, while it was rapidly released at higher pH values, thus allowing the drug to be selectively absorbed in the intestinal tract. These NPs were also proven to be non-toxic in a mouse fibroblasts model. Efficient biomedical applications could be accordingly envisaged for such an inclusion system [78].

Similar results were obtained for 3TC-CS NPs prepared by ionic gelation of CS with tripolyphosphate anions. These formulations offer several advantages with respect to conventional dosage forms of the drug, particularly in terms of bioavailability [79]. The CS functionalization with glycyrrhizin was realized for a liver targeting and a 3TC controlled release. In fact, the results of this research confirmed a lower drug release and an augmented level of 3TC in hepatocyte tissues, if compared with CS NPs or the free-drug solution [80]. Successively, 3TC was loaded into poly(ε-caprolactone) through a double emulsion spray-drying method giving rise to NPs with spherical morphology. This system also proved to be effective in improving drug bioavailability and reducing side effects [81]. Multiple drugs combined in a single nanosystem showed significant advantages over therapy based on a single drug. Accordingly, an example of polymeric NPs based on methyl methacrylate or ε-caprolactone was designed for the release of four different anti HIV drugs, AZT, 3TC, nevirapine and the IN inhibitor raltegravir [82]. In a recent paper, the incorporation of 3TC into CS with sodium alginate/calcium chloride by the above gelation method, in different experimental conditions, was described and showed an impressive drug release rate lasting up to 24 h. The method proved to furnish highly homogenous particles capable of improve bioavailability together with a constant drug release, following a first order mechanism, with diffusion of the drug after swelling of the polymer [83].

Protein-based NPs were prepared using lactoferrin for the controlled release of 3TC combined with AZT and EFV, after its application for a single DDS. The lactoferrin possesses itself antiviral activity and therefore acts synergistically with the entrapped drugs. The assessment of pharmacokinetic profile for each entrapped drug and *in vitro* data suggested that these NPs are able to release drugs intracellularly in a controlled and sustained manner [84,85].

Alternative nanotechnologies used for anti-HIV drugs release included the use of inorganic components such as iron or silica. Preliminary results suggested a potential application for these new formulations. In particular, chemical-physical characterization of SiO2 NPs coated with magnetic Fe_2_O_3_ loaded with 3TC were investigated for their pharmacokinetic and cytotoxic profiles showing more favorable features compared to the free drug [86].

A similar preparation was attempted for 3TC and zalcitabine both in form of triphosphosphates. Preliminary results showed that these nanosystems of dideoxynucleoside triphosphates/SiO2 NPs were useful transport systems for delivering these drugs to target cells with increased antiviral efficiency [87].

Moreover, the co-encapsulation of 3TC and AZT, both in form of triphosphates, into iron carboxylate mesoporous NPs gave biocompatible systems endowed with peculiar delivery properties. In particular, the drugs were released with different kinetics: 3TC showed accelerated release, while AZT was released more slowly. This vector protected drug from degradation, conferring at the same time improved *in vitro* anti-HIV activity. In fact, these formulations contribute to stabilizing the drugs since no alterations were detected after two-month storage and freeze-drying reconstitution [50]. High drug bioavailability and patient compliance were recorded after the administration of the of 3TC encapsulated into a new gum odina based biopolymer obtained by a multiple water-in-oil-in-water emulsion approach. The long-term stability study showed the improvement of the stability of the emulsions after a 90-day storage compared to a similar emulsion comprising Tween 80 as a stabilizer [88]. 3TC was also loaded in nanovesicles based on phospholipids or non-ionic surfactants (niosomes and liposomes). The best components and reparation methods able to produce formulations with suitable size, improved drug encapsulation efficiency and release profile were formulated for these systems [89,90]. 

### 3.4. Abacavir

Abacavir ((1S,4R)-4-(2-amino-6-(cyclopropylamino)-9H-purin-9-yl)cyclopent-2-en-1-yl)methan- ol, ABV) introduced in 1998, represented an alternative nucleoside derivative administered orally in solid or solution form for the prevention and treatment of HIV infection. Similarly to other nucleoside analogs, its use is recommended in combination therapy because of its severe side effects like hypersensitivity, liver damage and lactic acidosis, which all preclude monotherapy. ABV and its congener 3TC, after transformation into the corresponding thiol ending ester derivatives, were conjugated to glucose-coated gold NPs, which were investigated for their pH dependent drug release performances. This drug-delivery system was in turn studied for new multifunctional devices since such gold NPs, themselves endowed with microbicide properties, proved useful for the loading of more than one active agent differently targeting the viral replication cycle, and therefore representing a multivalent therapeutic approach [51]. Albumin NPs loaded with ABV sulfate were prepared by solvation method and studied for their mechanism of drug release. Results obtained revealed a remarkable drug loading capacity together with a sustained and controlled release within 24 h in HIV reservoir organs [91]. A myristoylated ABV prodrug entrapped into poloxamers was evaluated for the pharmacokinetic properties after injection in mice. Comparison of such nanoformulation with the free drug was performed on human monocyte-derived macrophages by proton nuclear magnetic resonance studies in terms of anti-HIV activity. 

As a result, an efficacy comparable to that of the native drug was detected for the encased polymer, which showed a two-week lasting release [92]. A detailed study described the formation of innovative nanocarriers named ProTide (PROdrug and nucleoTIDE) obtained by the loading of L-alanine and L-phenylalanine ester phosphoramidates of ABV into PLGA and poloxamer NPs. Such formulations showed sustained retention and antiretroviral activities for up to one month [93].

### 3.5. Emtricitabine 

Emtricitabine (4-amino-5-fluoro-1-((2S,5R)-2-(hydroxymethyl)-1,3-oxathiolan-5-yl)pyrimidin-2(1H)-one, FTC), is a deoxycytidine nucleoside analog approved in 2006 for anti-HIV therapy. Even if it showed reduced side effects when compared to other NRTIs, FTC is largely used in triple or quadruple drug combinations.

A customary PLGA nanoformulation of this water soluble drug was achieved through the water-in-oil-in-water emulsion method and showed a sustained release profile in rats, with adequate drug concentration up to two weeks [94,95]. The large volume distribution, beside a short plasma half-life, suggests the use of FTC in alternative formulations, such as PLGA NPs. This particular administration form proved to be able in enhancing drug stability and intracellular retention time, as demonstrated by an ex vivo endosomal assay. A once-biweekly dosing for HIV infection prevention or treatment was accordingly hypothesized [96]. In addition, eight degradation products were separated and characterized by LC–MS/MS from ABV sulfate when subjected to forced degradation under hydrolysis, oxidation, photolysis and thermal stressing conditions [97]. More recently, a solution state study showed the formation of eleven degradation products [98]. 

The thermal decomposition of FTC was well investigated by applying different methods. FTC largely decomposed to small molecules and insoluble substances. A small amount decomposed to 5-fluorocytosine due to an oxidation reaction [99]. When the drug was exposed to the action of acids or bases as well as oxidative stress conditions, an additional degradation product was detected [100].

### 3.6. Tenofovir

Tenofovir disoproxil fumarate ((R)-(((((1-(6-amino-9H-purin-9-yl)propan-2-yl)oxy)methyl) phosphoryl)bis(oxy))bis(methylene) diisopropyl dicarbonate, TDF) is a more recently approved NRTI (2001) used in the treatment of chronic hepatitis B and in the prevention and treatment of HIV infection. Successively its prodrug tenofovir alafenamide fumarate ((isopropyl 2-((((((R)-1-(6-amino-9H-purin-9-yl)propan-2-yl)oxy)methyl)(phenoxy)phosphoryl)amino)propanoate, TAF) was laun-ched in the market due to its more favorable properties after oral administration. TAF has greater antiviral activity and better distribution into lymphoid tissues than TDF. Both drugs are recommended in combination therapy along with other antiretroviral agents.

#### 3.6.1. Tenofovir SLN 

Lipid NPs loaded with NRTI and NNRTI agents including TDF or TAF were extensively studied for the improvement of bioavailability and long lasting drug release. Several lipid matrices were designed and showed a very promising behavior under different experimental conditions [37,101,102]. Toxic effects of TDF loaded in nanoemulsions on liver and kidney were assessed using an animal model. Although any behavioral toxicity and mortality were not detected, moderate alterations were however observed on both organs [103]. Extensive chemical-physical studies were performed on hybrid inclusion complexes obtained by encasement of TDF into lipid and polymer matrices by engineered melt emulsification-probe sonication technique. The carrier obtained by combining TDF, lauric acid and pemulen polymer was shown to promote a noteworthy increase of TDF trans-nasal flux, so potentially useful for nasal administration [104]. Nanocarriers based on a hydrogel-core and a lipid-shell were designed for the controlled loading and topical vaginal release of TDF and maraviroc, a virus entry inhibitor. These nanolipogels proved to be efficient systems and robust carriers for the encapsulation and the prolonged *in vivo* release of antiretroviral drugs, showing solubility concerns that are useful during the prevention and treatment of HIV infection [105]. 

#### 3.6.2. Tenofovir/Dendrimers Complexes

A drug combination including TDF into dendrimers was designed for the evaluation in an *in vitro* model of semen-enhanced viral infection. The results obtained suggested that this therapeutic strategy could bypass the detrimental effects of amyloid fibrils, present in semen, which seem responsible of the failure of topical vaginal gels action [54]. An approach to the treatment of neuro-AIDS was based on the use of co-encapsulated drugs into ultra-small iron oxide NPs with the addition of dextran sulfate. The inclusion complex of TDF and vorinostat, a latency-breaking agent, was assembled by magnetically guided layer-by-layer method and a noteworthy blood–brain barrier transmigration of drugs was then observed. This strategy, aimed to the activation of latent virus and its simultaneous killing, would result in a high efficacy to eradicate completely the infection from the CNS [106]. Nanosystems such as carbosilane dendrimers seem themselves able to inhibit HIV replication with a potential as local antiviral agents. Nevertheless, the concomitant administration of specific antiretroviral agents led to a potent synergistic activity. TDF, along with AZT and EFV or with maraviroc was encapsulated into anionic carbosilane dendrimers, bringing sulfated and naphthyl sulfonated groups to generate potential microbicides to prevent the sexual transmission of HIV [55,107,108,109,110]. An innovative therapeutic strategy could be based on the TDF prolonged release from NPs obtained by hyaluronic acid (HA) cross-linked with adipic acid dihydrazide. This nanosystem did not show detectable toxicity under the control of hyaluronidase enzyme. Comparative experiments with a simple TDF/HA gel suggested an essential role of the enzyme during the HA degradation and TDF release. The potential of these formulations for topical delivery of antiviral agents for the prevention of sexually transmitted diseases was accordingly hypothesized [111]. 

#### 3.6.3. Chitosan based TDF Nanoparticles

TDF was also used as a model drug in a CS based nanopreparation coated with sodium acetate, an aggregation-preventing agent, realized by the freeze-drying method. The NPs cytotoxic profile on macrophages was assessed by neutral red, resazurin, nitrite oxide and cytokines assays. Satisfactory encapsulation rate together with a good stability of the colloidal dispersions was observed for the formulation. Moreover, a sustained drug release beside a lack of cytotoxicity and a pro-inflammatory effect was recorded [112]. Further improvements in terms of mucoadhesive performance were obtained by a formulation based on TDF-loaded CS NPs dispersed in vaginal thermogels [113]. CS based oral NPs loaded with TDF were prepared by the ionic gelation technique and studied for their potential in preventing esterase metabolism and facilitate active transport uptake. Both processes were affected as confirmed by *in vitro* experiments. Moreover, data obtained suggested that a clathrin-mediated mechanism is involved in the enhancement of drug oral absorption [114]. A triple combination of TDF, FTC and bictegravir, an integrase inhibitor, was loaded into trimethyl CS to generate a nanoconjugate with improved cellular uptake. The efficiency of the nanocarrier was determined by spectrophotometry while XTT and ELISA tests were used to determine cytotoxicity and anti-retroviral efficiency, respectively. As a result, this formulation proved to inhibit viral replication at lower concentrations than the free drugs combination, without a significant cytotoxicity, therefore resulting in a lower drug resistance [115]. Colloids based on polyelectrolyte complexes of CS and chondroitin sulfate were loaded with TDF and examined for the stability at physiological conditions. This property was assured by the use of Zn(II) throughout the formulation procedure. *In vitro* studies did not reveal toxicity of such NPs on human peripheral blood mononuclear cells, while a remarkable dose-dependent antiretroviral activity was detected [116]. TDF was loaded into thiolated CS core/shell nanofibers in order to investigate the rate of drug loading, mucoadhesion properties and *in vivo* safety. The formulation was fabricated by assembling poly(ethylene oxide) with the CS component and PLA by a coaxial electrospinning technique. An enhanced drug loading together with a prolonged drug release and an increased mucoadhesion were assessed by in vitro studies, whereas a significant toxicity was not detected in neither *in vitro* nor *in vivo* experimental models. These new formulations could be therefore considered promising tools for the local delivery of microbicide agents [117]. 

#### 3.6.4. Alternative Polymeric TDF NPs

An original formulation to be used for vaginal administration was fabricated by oil-in-water emulsification of the inclusion product of TFV into PLGA and sodium deoxycholate as an ion-pairing agent and a thermosensitive gel. Sustained release properties in humanized BLT mice were shown for these nanoformulations when instilled locally [118]. Similar results were obtained by loading TFV into PLGA/stearylamine and incorporating such NPs into a hydroxypropyl methylcellulose/PVA-based film [119,120]. TAF and FTC entrapped NPs were prepared for subcutaneous administration during pre-exposure prophylaxis. Drugs were included into the PLGA/PVA system and investigated for their long-acting potency detectable even after 14 days by a humanized mice model [121,122]. A similar approach was exploited for the incorporation of TAF and elvitegravir, an integrase inhibitor, during the fabrication of devices to be used during vaginal prevention [123,124]. The drug absorption following oral administration were also positively affected by the use of TAF/PGLA loaded NPs, as highlighted by a statistical model study [125]. Formulations containing mono- or by-layered films of PVA and pectin were coupled with Eudragit NPs loaded with TDF/FTC, by nano spray-drying technique. These systems were designed for vaginal use with a better patient compliance. The time of disintegration and drug release was evaluated in a simulated vaginal fluid, showing favorable results. The by-layered films equipped with NPs loaded with drugs showed the best performances in terms of drug release delay. Moreover, this topic formulation was shown particularly safe by MTT and lactate dehydrogenase assays using different cervical cell lines [126]. Multifunctional magneto-plasmonic liposomes charged with TDF were obtained with the aim to study guided systems for enhancing efficiency of antiviral treatment. The distribution of such a hybrid system can be monitored by image technique and activated magnetically into the brain. The gold shell of such nanocomplexes can be followed by computed tomography. This way, these particular systems proved to be efficient against HIV in infected microglia cells after adequately crossing the BBB [127]. A nanosuspension of drug combination particles consisting of TDF, ritonavir and lopinavir, two protease inhibitors, and lipids were prepared for the development of innovative topical formulations. This system was highly efficient in targeting lymphocytes during anti-HIV therapy with a long-lasting action after a single subcutaneous administration [128]. Similar results were described after the addition of 3TC to the previously combination to give a four-drug components nanosuspension [129]. Similar results were obtained for alternative combinations of TDF and other RTI. In all cases, a persistent drug concentration was detected after single subcutaneous injection in different HIV reservoir cells [130,131]. A stability study was performed on TAF and compared with the stress degradation behavior of TDF. Gastrointestinal stability studies were conducted on both drugs, showing the formation of six degradation products. These studies revealed a higher stability of TAF, except for with the acid condition, where the drug was extensively degraded [132]. 

## 4. Non-Nucleoside Reverse Transcriptase Inhibitors Nanosystems

An alternative approach to anti-HIV treatment with RTIs is represented by the combined therapy using both NRTIs and NNRTIs, exploiting the synergism of the two distinct classes of drugs. Accordingly, the multi-therapy is nowadays the most widely adopted strategy for the treatment of HIV infection in the clinic. NNRTI act directly by binding the enzyme, so preventing its DNA polymerase function. In fact, their heterogeneous structures do not resemble those of nucleobases, the natural substrate of RT (Figure 4, panel c). 

**Figure 5:**
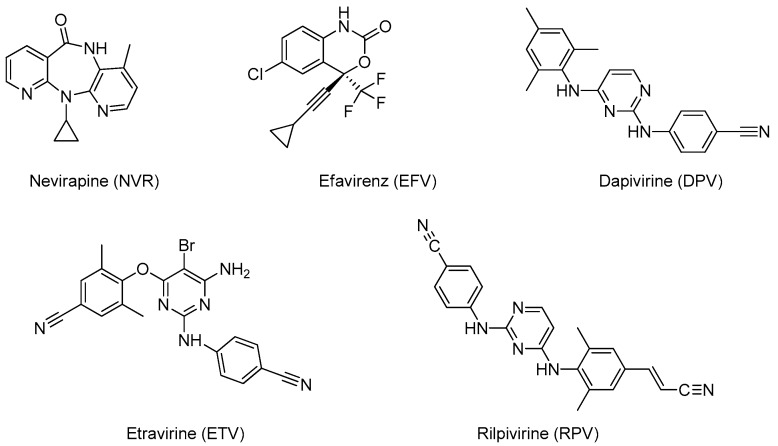
Chemical structures of NNRTI.

After a first generation NNRTI drugs introduced in the 90s (i.e. nevirapine, delavirdine, efavirenz) approved for anti-HIV therapy, recently some new and effective compounds entered the market (i.e. etravirine, rilpivirine). Some more interesting compounds are currently under clinical investigation. The structures of representative NNTRI are reported in Figure 5.

### 4.1. Nevirapine

Nevirapine (11-cyclopropyl-4-methyl-5H-dipyrido[3,2-b:2′,3′-e][1,4]diazepin-6(11H)-one, NVR) was the first NNRTI approved by FDA in 1996 for the treatment of HIV infection. In order to improve the pharmacokinetics of this hydrophobic drug, NVR was loaded into liposomes prepared by thin film hydration and extrusion method to give uniform spherical vesicles. The matrix, obtained from egg phospholipid and cholesterol, proved to release the drug during 22 h at physiological pH values. The presence of proteins into the medium or the exposition of the system to ultrasounds greatly impair the delivery mode of the drug. However, this encapsulation method could optimize the efficacy of NVR in terms of drug stability and controlled release to the target tissues [30]. Transferrin grafted PLGA NPs have been designed in order to facilitate NVR in crossing vascular endothelial cells of the human brain. This particular nanosystem allowed a favorable drug loading with a desired controlled release, so proving to act as an efficient carrier to promote vascular diffusion of the compound [133]. Nanoparticles of PLA/PEG, the surface of which was modified with serum albumin, were prepared to improve release of the drug to the target tissues. This favorable feature was measured after i.v. injection in rats, showing an improved bioavailability, cellular uptake and drug accumulation in the brain, liver and spleen, compared to pure drug solution or uncoated nanoformulations. Moreover, no additional cytotoxicity was recorded. The capability to cross the BBB makes these formulations potentially useful for the treatment of AIDS related dementia [134,135]. The stability of NVP was well investigated by a stability-indicating ultra-high performance liquid chromatography (UHPLC) method. Drug product efficacy, safety and quality were verified in different degradation conditions by using acids, bases, water, metal ions, heat, light and oxidation agents. The tests were applied on the pure compound and on its tablet formulation leading to the formation of five degradation products [136]. A physically stable formulation of NVP was prepared by forming a crystalline inclusion complex with biodegradable and hydrophobic poly(ε-caprolactone). Compared to pure NVP crystals, the formulation assured a sustained drug release at physiological conditions in PBS solution up to 6 weeks, due to the reduction of drug solubility [43].

### 4.2. Efavirenz

Efavirenz, ((S)-6-chloro-4-(cyclopropylethynyl)-4-(trifluoromethyl)-1H-benzo[d][1,3]oxazin-2(4H)-one, EFV) was approved in 1998 and is largely utilized with other drugs for anti-HIV association therapies. However, its potential was limited by a low bioavailability due to its high lipophilicity and other drawbacks related to irritant effects on mucosae [137,138]. A strategy devoted to the improvement of EFV pharmacokinetics resides in its incorporation into SLN, which should drive the delivery of the drug next to the lymphoid system and brain [38]. Phenylalanine anchored SLN (PA-SLN) were used to encapsulate EFV and the resulting nanocomplex was tested for its potential to cross BBB. Phenylalanine was chosen in order to exploit the aromatic amino acid transporter active within the barrier. The nanocomplex showed good entrapment efficiency and a favorable drug release, with a remarkable accumulation in brain assuring a long-lasting therapeutic effect [139].

#### 4.2.1. Efavirenz SLN

SLN of selected lipids as matrix medium and EFV were prepared with the addition of a surfactant by high-pressure homogenization technique and evaluated *in vivo* for their enhanced bioavailability and brain targeting. In particular, such properties were assessed after an intranasal administration route, which could be useful for therapy devoted to the complete eradication of HIV [39]. As an example, EFV was loaded into SLN assembled with the use of mono- and tri-glycerides with the aid of a surfactant. This particular formulation allowed the drug to partly by-pass liver metabolism after oral administration and in doing so increasing bioavailability and accumulation into spleen [140]. A prolonged drug release together with a lower incidence of side effects, consequent to a reduced drug dosage, was achieved after EFV incorporation into SLN. 

#### 4.2.2. Polymeric EFV NPs

Efavirenz was loaded into NPs based on methacrylate polymers, which conferred an increased drug uptake in monocytes and macrophages [141,142]. Emulsion or nanoprecipitation methods allowed loading EFV into biodegradable PLGA NPs. The effects of the resulting formulations administered in combination with other free or encapsulated anti-HIV drugs were investigated. As a result, the new formulations proved more efficient compared to the free drugs and also a noteworthy synergistic effect was recorded for encapsulated EFV combined with TDF, a second NRTI [143]. EFV was dispersed with α-tocopherol polyethylene glycol succinate and PVA by an emulsion-templated freeze-drying technique to realize solid inclusion NPs. Dry monoliths charged with these NPs proved to be stable for several months. Their reconstitution in water furnished nanodispersions, which showed reduced cytotoxicity together with an improved bioavailability and pharmacokinetics [49]. A nanoformulation was obtained by combining EFV with cellulose acetate phthalate, acting as an HIV entry inhibitor, by the nanoprecipitation method. The resulting NPs were formulated into a thermosensitive gel, which resulted in an efficient nanomicrobicide for long-term HIV prophylaxis [144]. Polymeric micelles based on pluronic F127 loaded with EFV and bio-conjugated with anti-M-cell-specific antibodies were prepared in order to evaluate their preferential target delivery to gut micro fold cells of lymphoid tissue, one of the major HIV reservoirs in the body. The efficiency of such nanosystem was showed to be higher than the free drug and a possible oral application by enteric-coated capsule was accordingly hypothesized [145]. EFV was loaded into eudragit, pluronic and alginate sodium polymeric based NPs by solvent evaporation method. Cytotoxicity and antiviral characterization was investigated by syncytium inhibition assay. The polymeric nanocarrier was proven to be more effective than the pure free drug by an enhanced drug dissolution and bio-distribution, especially after BBB crossing. A reduced toxicity was also detected [146,147]. NPs to be used during intranasal administration were prepared by the encasement of EFV into CS grafted hydroxypropyl beta cyclodextrin matrices. The results obtained confirmed a better CNS access due to an improved cellular permeation consequent to both a higher drug solubility and a concomitant beneficial action of CS on cell membrane [148]. Rectal polymeric formulations were prepared by incorporation of EFV into PLGA NPs coated with PEG. This particular form assured a prolonged drug residence in the lower colon with a long lasting prophylactic action against HIV transmission [149]. A better BBB crossing together with a reduction of side effects incidence was attempted by the preparation of transferrin functionalized PLGA NPs loaded with EFV. Although a higher deposition rate of the drug to the targeted site was recorded, the formulation did not improve drug membrane permeation [150]. Lactoferrin NPs were fabricated for oral or vaginal administration giving raise to significant results in terms of drug release and bioavailability. A combination of EFV and curcumin was also attempted in order to reach synergism for the two-microbicide agents. The results obtained confirmed an improved pharmacokinetic profile for the drug combination nanoformulation with respect to free drugs [151,152]. 

#### 4.2.3. Efavirenz/Dendrimer Complexes

EFV was incorporated into t-Boc–glycine and mannose conjugated dendrimer-based poly(propyleneimine) (PPI). These branched three-dimensional polymers were shown to be less toxic than free PPI and could allow the drug to reach monocytes and macrophages, both reservoirs of HIV in the body. In fact, it is known that inhibitors targeting only lymphocytes are ineffective in completely eradicating the infection. These formulations demonstrated to be particularly effective since they are able to promote a significant increase of EFV cellular uptake [153]. The same authors loaded EFV into dendrimer-based PPI complexed with tuftsin (Tu). This latter is a tetrapeptide coming from the cleavage of immunoglobulin G and showed to be capable of selectively target and activate macrophages, monocytes and polymorph nuclear leukocytes. This strategy would help EFV in avoiding unwanted effects acting in a synergistic fashion with the peptide. Such an option was suggested by the fact that the TuPPI complex showed no toxicity and prolonged EFV cellular uptake in HIV infected macrophages with respect to uninfected cells [154]. 

#### 4.2.4. Alternative Supramolecular EFV NPs

Vesicular systems consisting of gold NPs entrapped inside the aqueous core were loaded with EFV in the bilayer membrane. The niosomes so formed were dispersed in carrageenan/hyaluronic acid/poloxamer based thermogel after coating with an opportunely functionalized mannose, in order to combine the action of the protein and the sugar for an effective prophylactic vaginal application. A remarkable inhibition of viral transmission as well as a drastic reduction of side effects was recorded [31]. A soya lecithin/cholesterol and PEG liposomal DDS was designed in order to overcome the limited solubility, dissolution rate and bioavailability of EFV [32]. Similar results were achieved by the entrapment of EFV into boron nitride and carbon nanotubes as delivery vehicles. Both the systems showed a favorable behavior. Between the two distinct formulations, carbon nanotubes assured a higher drug adsorption [155]. Enhanced solubility and dissolution of the drug were also obtained by inclusion complex with hydroxypropyl-β-cyclodextrin. The solubility of the complex was further increased by the addition of l-Arginine [156]. Degradation behavior of the drug in water solution was assessed by HPLC analyses, detecting a total twelve degradation products under acidic conditions. Alkaline stress resulted in the formation of only two degradation products, whereas oxidative and photolytic conditions did not promote any degradation of the drug. All the degradation derivatives showed remarkable toxicity, including carcinogenicity, mutagenicity and skin irritation, as confirmed by *in silico* experiments [72]. The thermal behavior of glass EFV was evaluated in different experimental conditions, showing a good stability only at room temperature [157].

### 4.3. Dapivirine

Dapivirine (4-((4-(mesitylamino)pyrimidin-2-yl)amino)benzonitrile, DPV) is a new, sparingly soluble NNRTI, under investigation for vaginal application during the prevention of HIV sexual transmission. This compound was shown to be a noncompetitive inhibitor of RT that is particularly useful for topic treatments. In order to improve efficacy, a microbicide film for vaginal delivery was formulated by loading DPV into PLGA NPs and the nanosystems were in turn coupled to a PVA in a cellulose based platform film using solvent casting technique. TDF was also added to the formulation to achieve a synergist antiviral effect. A rapid release of active principles was accordingly recorded suggesting an ideal behavior of such a formulation as prophylactic microbicide [158,159]. Some advantages were also obtained by alternative formulations of this drug. In particular, surface-engineered poly(ε-caprolactone) NPs were manufactured and evaluated in pig vaginal and rectal mucosa, resulting in favorable drug release properties [160]. Physical–chemical properties of these nanocarriers were evaluated upon one-year storage to a variable temperature range. Colloidal instability affected the *in vitro* drug release of the NPs, although no detectable degradation was observed for the entrapped drug [56]. 

### 4.4. Etravirine

Etravirine (4-((6-amino-5-bromo-2-((4-cyanophenyl)amino)pyrimidin-4-yl)oxy)-3,5-dimethyl-benzonitrile, ETV) is a NNRTI approved in 2008 for monotherapy against HIV, also clinically used in combination with other antiviral agents in antiretroviral treatment-experienced adult patients with onset of resistance. A combination of ETV, maraviroc and raltegravir was loaded into PLGA by emulsion-solvent evaporation technique. The antiviral potency of the resulting NPs was compared to the free triple drug combination in an *in vitro* cells assay and on a macaque cervicovaginal explant model *in vivo*. The nanoformulation provided a prolonged release by extension of the half-life of drugs, leading to an enhanced synergistic antiviral action [161]. An innovative approach for the design of NPs overcoming the drawback, consisting in low drug retention and massive leakage, was undertaken by the preparation of NP-releasing nanofiber vaginal devices. Mucoadhesive PVA fibers coupled to PEGylated NPs should ensure an adequate retention together with a rapid mucus diffusion. These composite nanoformulations proved to assure a sustained ETV release for up to seven days [162]. 

### 4.5. Rilpivirine

Rilpivirine ((E)-4-((4-((4-(2-cyanovinyl)-2,6-dimethylphenyl)amino)pyrimidin-2-yl)amino) benzonitrile, RPV) is a second-generation NNRTI, approved in 2011, possessing increased potency, longer half-life and lesser side-effects with respect to former non-nucleoside agents, today mainly used in combination with other anti-HIV drugs [163]. RPV met therapeutic success either in combination with other anti-HIV agents or in long-acting injectable nanoformulations during maintenance therapy. Its use could also be advantageous for the prophylactic treatment of high-risk uninfected individuals [164,165]. In two separate papers, the potential use of long acting RPV NPs associated with cabotegravir, an HIV integrase inhibitor, was described and an innovative monthly dosing therapeutic regimen was accordingly proposed. Recently, the result of a phase IIb study was reported on the effectiveness of this combination nanoformulation in maintaining adequate drug concentration in plasma or vaginal mucus for up to one month [166,167,168]. RPV-loaded PLGA NPs were fabricated by emulsion-solvent evaporation method. A sustained release in plasma as well as faster clearance were observed in animal models, suggesting a prophylactic use after the preparation of thermosensitive gels as well as long acting injectable nanosuspensions [169].

Biodistribution of tri-modal theranostic NPs was studied by single-photon emission computed tomography, magnetic resonance imaging and fluorescence techniques. These devices were prepared by the incorporation of Indium radiolabeled, europium doped cobalt-ferrite particles and RPV loaded into a poly(ε-caprolactone) matrix included into a lipid shell. A sustained drug release and antiretroviral activity were observed in HIV infected macrophages. These multi-functional NPs represent a platform for the monitoring and optimization of the antiretroviral drug pharmacokinetic profile [170]. 

## 5. Conclusion

There is a high level of interest in nanotechnologies for their relevant roles in the design and development of innovative anti-HIV formulations either for oral or topical administration. In the first case, a more specific targeted delivery together with a sustained release represents the major goal in the field. Mucoadhesive performance, prolonged retention time and improved patients’ compliance were desired for vaginal or rectal application of microbicide nanoformulated antiretroviral agents. A large number of diversely assembled nanocarriers loaded with different classes of antiviral drugs were then developed and deeply investigated to improve both pharmacokinetic characteristics and stability behavior. Overall results demonstrated a potential success of such an approach for monotherapy, even though more profitable applications were recorded for the combination therapy, the most diffused method nowadays. In fact, anti-HIV therapeutic regimens today comprise multiple daily doses of more drugs acting at different stages of viral replication, which lead to poor patient compliance. Although the use of protease, integrase and viral entry inhibitors has become customary, NRTIs and NNRTIs remain pivotal tools during the infection management. In the light of these findings, this overview focuses mainly on the application of nanotechnology applied to RTI as vehicles for challenging virus eradication from depot organs and/or as a platform for the design of modern prophylactic devices for stable or stressing conditions. 

## Figures and Tables

**Table 1:** Relevant information of currently used RTI. *Year of FDA approval; LS = Lipid Solubility; OB = Oral Bioavailability; t/2 = Plasma Half-life.

Drug Class	Name (Acronym)	Year*	LS	OB (%)	t/2 (hours)	Side Effects
NRTIs	Stavudine (STV)	1996	low	86	1.3–1.4	Peripheral neuropathy, pancreatitis, asymptomatic acidosis, lipoatrophy, hepatic steatosis
	Zidovudine (AZT)	1986	low	60	0.5–3	Neutropenia, anemia, nausea, vomiting, asthenia, headache, insomnia, skin hyperpigmentation, acidosis, hepatic steatosis
	Lamivudine (3TC)	1995	low	86	5–7	Cough, diarrhea, fatigue, headache, malaise, nasal symptoms, lactic acidosis, hepatic steatosis
	Abacavir (ABV)	1998	low	83	0.8–1.5	Systemic respiratory hypersensitivity, gastrointestinal symptoms, fever, tiredness, sore throat
	Emtricitabine (FTC)	2006	low	93	8–10	Headache, nausea, upset stomach, diarrhea, trouble sleeping, dizziness, skin rash, strange dreams, cough, runny nose
	Zalcitabine (ZCT)	1992	low	85		Peripheral neuropathy, stomatitis, esophageal ulcerations, acidosis, hepatic steatosis
	Didanosine (DDN)	1991	low	30	2	Gastrointestinal intolerance, peripheral neuropathy, pancreatitis, asymptomatic acidosis, lipoatrophy, hepatic steatosis
	Tenofovir (TDF)	2001	low	25-39	12–15	Nausea, depression, confusion, headache, hitching, weakness, kidneys problems
NNRTIs	Nevirapine (NVR)	1996	moderate	92	25–30	Rash, Stevens-Johnson syndrome, elevated transaminases blood level, hepatitis, severe hypersensitivity reaction
	Efavirenz (EFV)	1998	high	50	40–55	Rash, Stevens-Johnson syndrome, sleep disturbances, dizziness, vertigo, depression, euphoria, difficulty concentrating, hallucination.
	Etravirine (ETV)	2008	high	--	30–40	Rash, Stevens-Johnson syndrome, toxic epidermal necrosis and multiform erythema, hypersensitivity reactions, hepatic failure
	Rilpivirine (RPV)	2011	high	50	19	Rash, depression, liver problems, mood changes

**Table 2:** Targets of DDS designed for anti-HIV therapy.

DDS		TARGET
Matrix	Surface	
Liposome NPs	Mannose	Liver, spleen, lung, brain, macrophages
Liposomes	Galactose	Liver
Chitosan NPs	Glycyrrhizin	Liver
NPs	Transferrin	Brain, endothelial cells
NPs	Serum albumin	Brain, liver, spleen
SLN	Phenylalanine	Blood brain barrier
Polymeric micelles	Anti-GP2 antibody	M-cell of gut-associated lymphoid tissue
Dendrimers	Tuftsin	Macrophages, monocytes, polymorph nuclear leukocytes

**Table 3:** Advantages and limitations of anti-HIV DDS.

DDS	Advantages	Limitations
**Liposomes**	Co-delivery of hydrophilic and lipophilic drug Selective uptake by mononuclear phagocytes Surface modification with target moiety of virus reservoir	Low drug loading capacity Physical and chemical instability Drug leakage Difficulty in sterilization Short half-life Poor scale up
**Niosomes**	Chemical stability Protection of drug from degradation Large uptake by mononuclear phagocytes and localization in virus reservoir organs Less expensive respect liposomes Functionalization with target ligand	Physical instability during the storage Difficulty in sterilization Difficulty in large-scale production
**Polymeric NPs**	High drug loading capacity Co-delivery of different drug for anti-HIV combination therapy Selective uptake by lymphoid organ Prolonged circulation time Surface functionalization with target moiety	Fast burst release Limited safe correlated to polymer toxicity High cost production
**SLN**	Higher stability and biological compatibility than liposomes and polymeric NPs Increase the bioavailability of poorly water soluble drug Avoidance of organic solvent Slow uptake by the RES Feasible-large scale production and sterilization Less expensive than polymeric and surfactant carriers	Low drug solubility in lipid matrix and loading capacity Drug leakage Particle growth Unpredictable gelation tendency
**Dendrimers**	Uniform particle size Large surface functional groups for the conjugation with target moieties	Toxicity problems
